# Heterogeneous responsiveness to environmental stimuli

**DOI:** 10.1093/beheco/araf023

**Published:** 2025-08-16

**Authors:** Jerome Cavailles, Christoph Kuzmics, Martin Grube

**Affiliations:** Institute of Biology, Division of Plant Sciences, University of Graz, Holteigasse 6, 8010 Graz, Austria; Department of Economics, University of Graz, Universitätsstrasse 15/F4, 8010 Graz, Austria; Institute of Biology, Division of Plant Sciences, University of Graz, Holteigasse 6, 8010 Graz, Austria

**Keywords:** behavioral ecology, equilibrium, ESS, individual differences

## Abstract

Individuals of a species cope with environmental variability through behavioral adjustments driven by individuals’ responsiveness to environmental stimuli. Three key empirical observations have been made for many animal species: The *coexistence* of different degrees of responsiveness within one species; the *consistency* of an individual’s degree of responsiveness across time; and the *correlation* of an individual’s degree of responsiveness across contexts. Taking up key elements of existing approaches, we provide one unifying explanation for all three observations, by identifying a unique evolutionarily stable strategy of an appropriately defined game within a stochastic environment that has all three features. Coexistence is explained by a form of negative frequency dependence. Consistency and correlation is explained through potentially small, individual, differences of states animals have and the resulting differential advantages they can get from it. Our results allow us to identify a variety of testable implications.

## Teaser

Animals within the same species often respond differently to environmental changes. Individual behavior, however, is quite consistent across time and contexts. Yet, there seem to be no identifiable characteristics that could explain these differences in behavior. Using a formal game-theoretic model of animal foraging, we show that persistent and consistent differences in behavior can arise as a stable mix of strategies even when there are only the slightest differences in the animals’ personal preferences.

## Introduction

Individuals generally cope with frequent changes in their environment (Candolin et al. 2023) by means of behavioral adjustments, see eg Coulson et al. (2017), Sih et al. (2012), Wong and Candolin (2015), Sih (2013). To be able to adjust their behavior to changes in the environment, individuals need to be responsive to environmental stimuli, observable indicators of these changes (Stamps (2016) refers to “[t]he extent to which the phenotype of an agent varies as an immediate response to variation in external stimuli” as “contextual plasticity”).

It is well documented that individuals differ in the degree of responsiveness to external stimuli, a phenomenon sometimes referred to as behavioral plasticity, see eg Mitchell and Houslay (2021), Laskowski et al. (2022) (to facilitate the required responsiveness even a single genotype can often display a range of phenotypes, a phenomenon referred to as a reaction norm, a concept that originates from Woltereck (1909)). More generally, *phenotypic plasticity*—which use the alterations in an organism’s behavior, morphology, and physiology as a response to its specific environmental conditions—have been extensively studied. Phenotypic plasticity was once undervalued and largely overlooked as a mechanism and concept of evolution. However, a shift in perspective is occurring due to recent theoretical and empirical studies. These studies highlight the importance of plasticity in fostering novelty and diversity across morphological, physiological, behavioral, and life history traits. For recent overviews, see Pfennig (2021) and Sommer (2020).

This difference in environmental responsiveness constitutes the main characteristic of personalities, and personalities have been observed in more than 100 species, see eg the survey by Nettle and Penke (2010). In a broader sense, animal personality can be related to plasticity (Dingemanse et al. 2010). Three key observations have been made for many animal species, as, for instance, highlighted by Wolf et al. (2007), see also Bell et al. (2009): The *coexistence* of different degrees of responsiveness within one species; the *consistency* of an individual’s degree of responsiveness across time; and the consistency, often referred to as *correlation*, of an individual’s degree of responsiveness across contexts (the “suite of correlated behaviors [...] reflecting the individual consistency across [...] situations” has been referred to as a “behavioral syndrome” by Sih et al. (2004)).

A few theoretical approaches explain one or more of these three observations. The theory of biological sensitivity to context, as in Pluess (2015), Boyce (2016), and Ellis and Boyce (2008) explains the coexistence of different degrees of environmental responsiveness with differences in individuals’ experiences in their early development. The theory of differential sensitivity, as in Ellis et al. (2011), interprets the difference of behaviour as a way to hedge future generations against the uncertainty in the environment, recently formalized by Bergstrom (2014) and Frankenhuis et al. (2016). Pluess (2015), also Mitchell and Houslay (2021), argue in favor of an integration of the two theories. A third approach is built around the idea of “negative frequency dependence”: The more individuals are responsive to environmental stimuli the less the benefits of being responsive. Negative frequency dependence is a cornerstone for explanations of the coexistence of different degrees of environmental responsiveness in the seminal models of Wolf et al. (2008), Wolf et al. (2011), and Wolf and McNamara (2012). See Dingemanse and Wolf (2010)  for a review of earlier models. While negative frequency dependence is able to explain coexistence, consistency and correlation are often explained by an individual’s state (eg morphology, phenotype, size, etc.), as in Wolf and Weissing (2010) and Dingemanse and Wolf (2010). Wolf et al. (2008), Ehlman et al. (2022) explain consistency and correlation with a “positive-feedback mechanism”: responsiveness is less costly for individuals that have been responsive before. Wolf and McNamara (2012) explain consistency and correlation by small variations of individuals’ metabolism (which is a form of state). However, the meta analysis by Niemelä and Dingemanse (2018) shows a weak link between state and personalities (individuals’ state can only explain between 3% and 8% of the personality differences). One of the main points of this paper is that we can reconcile this seeming contradiction by showing that even very small and possible non-measurable differences between individuals’ (internal) state can explain large differences in behavior.

The objective of this paper is twofold. First, we aim to provide a unified explanation of these three findings. We do this by building a stylized game-theoretic model in which there is a unique evolutionarily stable strategy that has the three properties. For concreteness we chose the specific setting of foraging from multiple food sources, which allows us to additionally build on the existing game-theoretic literature on the *ideal free distribution* of Fretwell and Lucas (1969): individuals allocate themselves proportionally to the amount of food available at each food source. This distribution has been shown to be an Evolutionarily Stable Strategy (ESS) (Cressman and Křivan 2006, Cressman et al., 2004). For a game-theoretical review, refer to Křivan et al. (2008). Second, we derive potentially testable implications from this model in terms of how the frequency of responsive individuals changes with changes in the environmental parameters. Finally, we provide a few extensions to assess the robustness of our findings with respect to some of the simplifying assumptions.

The rest of the paper proceeds as follows. Section 2 provides the basic game-theoretic model. Section 3.1 provides the unique symmetric Nash equilibrium of the class of games under consideration. This equilibrium exhibits “coexistence” of different degrees of environmental responsiveness. Section 3.2 provides a further discussion of the implications of equilibrium behavior, while Section 3.3 discusses the evolutionary stability of the equilibrium. Section 3.4 then extends the model slightly to accommodate both “consistency” and “correlation” of individuals’ degree of responsiveness, across time and contexts, respectively. Section 3.5 considers the case of noisy information, and Section 3.6 provides a generalization of the basic model to general distributions of food availability at the stochastic source. Section 4 finally concludes with a further discussion of the results and further related literature. The online Appendix provides some of the more technical arguments behind some of the results in the paper.

## Methods

In this section, we present the simplest possible model of interest for our problem. The key parameters and their definitions are summarized in Table 1. Each of n<∞ individuals can go to one of two food sources A or B. Food source A has a fixed amount of food normalized to n units of nutrition to facilitate an easier comparison when we vary the number of individuals n. Food source B has a stochastic amount of food n⋅X, with X drawn from a binary distribution with X=η with probability α and X=λ with probability 1−α with λ denoting a low and η a high food-availability at the stochastic food source B. We assume that $\frac{1}{n}<\lambda <\eta <n$. If the number of individuals n is large, the assumption represents barely a restriction. If n is not very large, the assumption represents the most interesting case. Suppose, for instance, that both $\lambda ,\eta <\frac{1}{n}$. This would imply that, the food-availability at the stochastic source B is always less than 1, while the food-availability at source A is n. Thus, as we are assuming equal food-sharing (see below), no individual would ever benefit from going to the stochastic food source. Even if all individuals go to food-source A an individual who switches to the stochastic source B gets a food-share of 1 only. Suppose, as another extreme, that both λ,η>n. Then the food-availability at the stochastic source B always exceeds $n^{2}$ and, therefore, no individual would ever benefit from going to the fixed source A. They get more than n units of nutrition at food source B and only at most n at food source A.

Before making their choice of food source, individuals can, in principle, inform themselves about the state of food source B at an arbitrarily small cost *c* > 0; individuals can choose to learn whether X=λ or X=η. If uninformed they can then choose to go to food source A or B. If informed they can react to the information in one of two ways. They can be, what we term, *responsive* by going to A when X=λ and going to B when X=η, or, what we term *counter-responsive* by doing the opposite. We denote the set of strategies by S={A,B,R,C}, for always going to food source A, always to B, being responsive, and being counter-responsive, respectively.

**Fig. 1.**
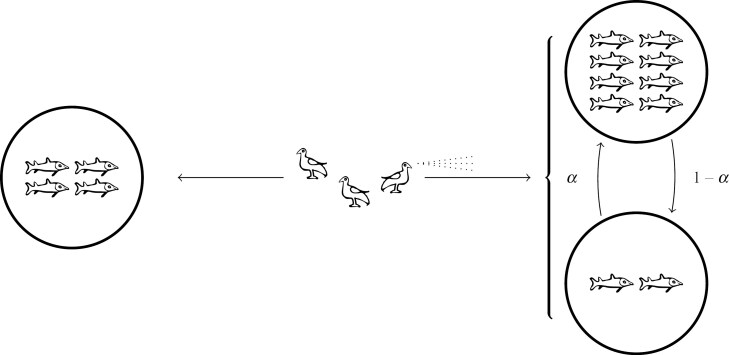
A graphical sketch of the basic model. The birds are the players, who choose which food source to go to: fixed food source A on the left or stochastic food source B on the right, with α the probability of high food availability. The bird’s scanning indicates that players can make their decisions based on observing the food availability at the stochastic source. In this concrete example, at the equilibrium, one bird should always go to the left, one bird should always go to the right, and one bird should be responsive to change. They will all eat two fishes when the stochastic source is low, and four fishes when the stochastic source is high.

The basic model (provided in Section 2) is sketched in Fig. 1. To illustrate the model we use fish-feeding birds as an example (inspired from observations of Wakefield et al. (2015), McHuron et al. (2018), Harris et al. (2020), Patrick et al. (2014)), with the simplifying condition that they do not show any social behavior (such as flocking or swarming).

**Table 1.** Overview of key notation.

Symbol	Description	Range
	Parameters	
n	number of individuals	N
λ	lower amount of food at the variable food source	$R^{*+}$
η	higher amount of food at the variable food source	$R^{*+}$
α	probability to have a high amount of food at the source B	[0, 1]
c	cost of gaining information	$R^{+}$
X	Stochastic variable of the amount of food at the source B	{λ,η}
	Strategies	
S	Set of strategies	
A	Strategy *always go to food source A*	
B	Strategy *always go to food source B*	
R	Strategy *being responsive*	
C	Strategy *being counter-responsive*	

Let Δ(S) denote the set of all mixed strategies, that is the set of all probability distributions over S. There are two ways to interpret a mixed strategy σ∈Δ(S), both go back at least to Maynard Smith and Price (1973) who speak both of the *probability* and the *frequency* of certain actions. One can think of a mixed strategy as a way an individual randomizes over pure strategies. These pure strategies are then played with the probability specified by σ. But one can also think of a mixed strategy as resulting from a random draw of a large (essentially infinite) population of individuals with given proportions of them playing the various pure strategies. These pure strategies are then present in the population with frequencies given by σ. While mathematically equivalent, this distinction matters for how our results are able to explain both the consistency and correlation of behaviour, which we investigate in the respective section below.

We assume throughout the paper that all individuals, who go to the same food source, share the available food at this source equitably. The payoff to an individual then only depends on the number of other individuals, k, that go to food source A (which implies that n−1−k others go to food source B). The payoff to an individual who goes to food source A is given by $\frac{n}{k+1}$; the payoff to an individual who goes to B is given by $\frac{nX}{n-k}$.

Given that individuals can choose mixed strategies, we need to compute individuals’ expected payoffs. To do so, consider an arbitrary individual who is facing that all other n−1 individuals choose a given (mixed) strategy σ∈Δ(S) with σ(s) the probability that pure strategy s is chosen. Below is presented the payoff for n→∞. See online Appendix 6 for the general treatment for any number of players n.


$$\begin{array}{l} \begin{array}{lllllllllllllllllllllllllllllll} u\left(A,\sigma \right) & = & \alpha \frac{1}{\sigma \left(A\right)+\sigma \left(C\right)} & + & \left(1-\alpha \right)\frac{1}{\sigma \left(A\right)+\sigma \left(R\right)} & & \;u\left(B,\sigma \right) & = & \alpha \frac{\eta }{\sigma \left(B\right)+\sigma \left(R\right)} & + & \left(1-\alpha \right)\frac{\lambda }{\sigma \left(B\right)+\sigma \left(C\right)} & & \;u\left(R,\sigma \right) & = & \alpha \frac{\eta }{\sigma \left(B\right)+\sigma \left(R\right)} & + & \left(1-\alpha \right)\frac{1}{\sigma \left(A\right)+\sigma \left(R\right)} & - & c\;u\left(C,\sigma \right) & = & \alpha \frac{1}{\sigma \left(A\right)+\sigma \left(C\right)} & + & \left(1-\alpha \right)\frac{\lambda }{\sigma \left(B\right)+\sigma \left(C\right)} & - & c\; & & & & & & \; \end{array} \end{array}$$ (1)

To derive these equations we use a result from Chao and Strawderman (1972). To add some intuition behind these payoff expressions, consider for instance u(A,σ), the payoff an individual obtains when it uses strategy A (of going to food source A regardless of the food availability at source B) and all others use the mixed strategy σ. There are two possibilities: the amount of food available at the stochastic source B is either high (with probability α) or low (with probability (1−α)). If it is high, all individuals who chose either strategy A or C will go to food source A. A-strategists do so, because they do so regardless of the food availability at source B and C-strategists do so, because they react to a high food availability at B with the counter-responsive action of going to A. Thus, the total number of individuals who go to food source A is approximately n times the proportion of A- and C-strategists: σ(A)+σ(C). They all share n units of food, so their expected food-share is approximately $\frac{1}{\sigma \left(A\right)+\sigma \left(C\right)}$. It is exactly that if n→∞. Similarly, with probability (1−α) the food availability at source B is low, in which case A- and R-strategists go to A and share the food there. This explains the second term in the sum. The other payoff expressions derive from analogous arguments.

## Results

### Equilibrium

A necessary condition for a strategy σ to be an evolutionarily stable strategy (ESS) is that it is a symmetric Nash (1950) equilibrium (to be precise, we mean ESS in the sense of Palm (1984), see also Broom et al. (1997), who have extended the definition of an ESS of Maynard Smith and Price (1973) to symmetric n-player games). This means σ needs to satisfy that whenever any n−1 players use strategy σ the remaining player also wants to use σ, ie finds that σ yields a payoff that is at least as high as that of any other strategy (given all others play σ). See online Appendix 6 for a formal definition of a symmetric Nash equilibrium.

In online Appendix 6 we show that the game at hand, under the given assumptions, always has a unique symmetric Nash equilibrium. This unique equilibrium is also an ESS as we show in online Appendix 10 and asymptotically stable under many classes of evolutionary dynamics as we show in online Appendix 11.

For any number of players n≥2 and any cost of gaining information c>0 that is not too large, this unique equilibrium has the following properties: the frequency of individuals using the counter-responsive strategy C, σ(C) is zero; the remaining frequencies of individuals always going to the fixed food source A, σ(A), of individuals always going to the stochastic food source B, σ(B), and of responsive individuals, σ(R), are all positive.

In general, we cannot explicitly compute these equilibrium frequencies, but if we consider the limit case of the number of individuals going to infinity, n→∞ and the cost of gaining information going to zero, c→0, the unique solution is


$$\begin{array}{l} \sigma \left(A\right)=\frac{1}{1+\eta }, \end{array}$$

$$\begin{array}{l} \sigma \left(B\right)=\frac{\lambda }{1+\lambda }, \end{array}$$

and


$$\begin{array}{l} \sigma \left(R\right)=\frac{\eta -\lambda }{\left(1+\eta \right)\left(1+\lambda \right)}. \end{array}$$

In the following section we explore some implications of this finding.

### Equilibrium implications

**Fig. 2.**
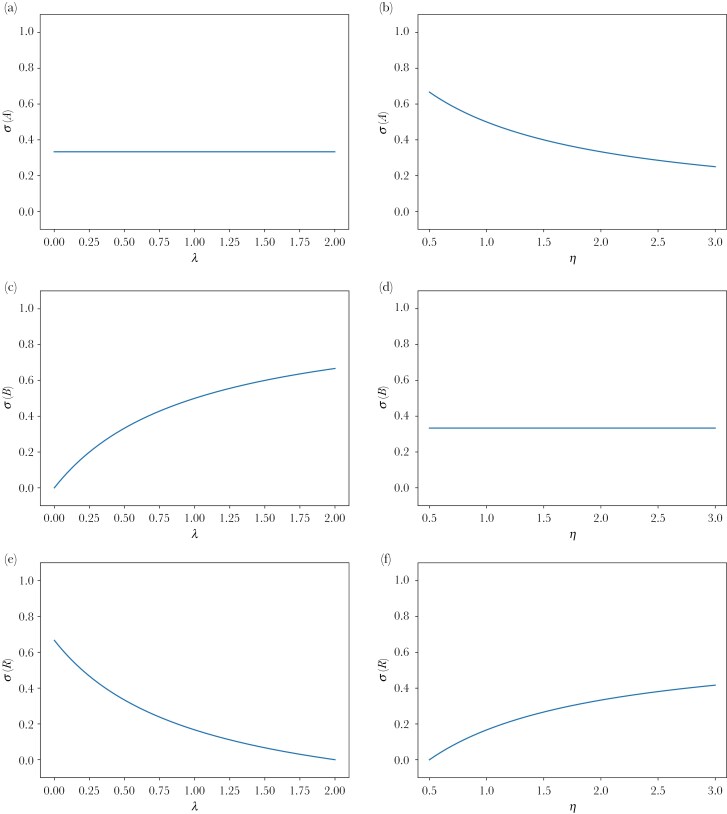
Equilibrium frequencies as a function of parameters: (a) σ (*A*) as a function of λ (with η = 2, in the limit case *c* → 0 and *n* → ∞), (b) σ (*A*) as a function of η (with λ = 0.5, in the limit case *c* → 0 and *n* → ∞), (c) σ (*B*) as a function of λ (with η = 2, in the limit case *c* → 0 and *n* → ∞), (d) σ (*B*) as a function of η (with λ = 0.5, in the limit case *c* → 0 and *n* → ∞), (e) σ (*R*) as a function of λ (with η = 2, in the limit case *c* → 0 and *n* → ∞), (f) σ (*R*) as a function of η (with λ = 0.5, in the limit case *c* → 0 and *n* → ∞).

Note first that the equilibrium frequency σ(A) of always going to the constant food source A does not depend on λ, the low state of food-availability in the stochastic source: σ(A) does not change when λ changes. This is not only true in the case of c→0 and n→∞, but generally true. This can be seen by the fact that Equation 4 in online Appendix 6 is an equation just in σ(A) (not also in σ(B) or σ(R)) and the parameter λ does not appear in this equation. Similarly, the equilibrium frequency σ(B) of always going to the stochastic food source B does not depend on η, the high state of food-availability in the stochastic source. Only the equilibrium frequency, σ(R) of being responsive changes with both levels of food-availability of the stochastic source. For a fixed high level of food-availability η at the stochastic source, increasing λ leaves σ(A) constant, increases σ(B), and decreases σ(R), the frequency of responsive individuals. Analogously, for a fixed low level of food-availability λ at the stochastic source, increasing η decreases σ(A), leaves σ(B) constant, and increases σ(R). All these findings are illustrated in Fig. 2. If both η increases and λ decreases (ie the stochastic food source becomes more extreme) then σ(A) and σ(B) decrease and, therefore, the equilibrium frequency of responsive individuals, σ(R) increases. Note also that it is possible that the difference, η−λ, between the high and low food availability at the stochastic source increases and yet the equilibrium frequency of responsive behavior, σ(R), decreases. To see this, suppose for a moment that both the high and the low food level (at the stochastic source) increase so that their difference remains the same. Then the equilibrium frequency of responsive behavior, σ(R), decreases (as the numerator remains the same and the denominator increases). This means that there are also situations where both the high and the low food level (at the stochastic source) increase so that their difference increases a little bit, and yet the equilibrium frequency of responsive behavior decreases.

The second thing to note is that in the limit in which the cost of gaining information becomes negligible (relative to the other parameters), that is when c→0 (for any n), the equilibrium frequencies do not depend on the stochastic nature of the environment, ie do not depend on α (this can be seen by setting c=0 in equations 4 and 5 in online Appendix 6, with the result that α drops out of these equations.

**Fig. 3.**
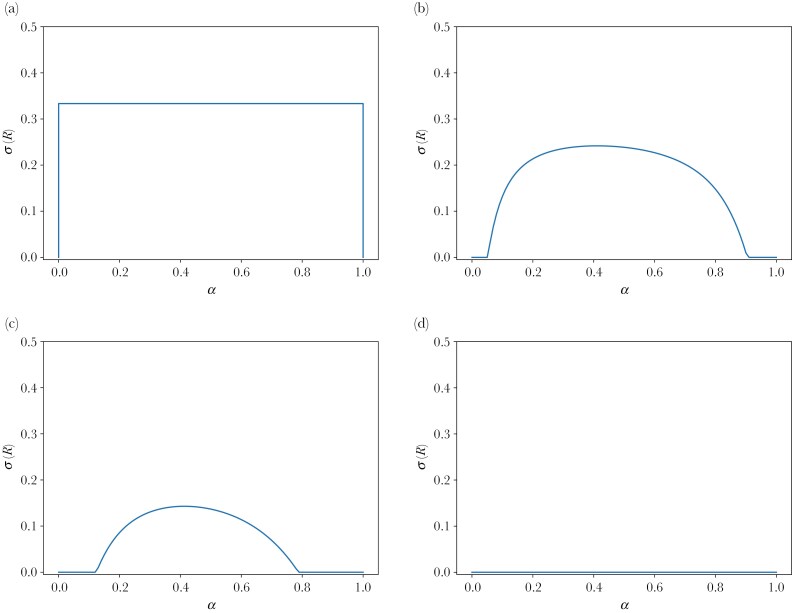
Probability or frequency of being responsive, σ(R) as a function of the probability, α of the high food availability at the stochastic food source, for different levels of the cost of gaining information, c (for λ=0.5,η=2). (a) Zero cost of gaining information, *c* = 0, (b) Low cost of gaining information, *c* = 0.2, (c) Higher cost of gaining information, *c* = 0.4, (d) Prohibitive cost of gaining information.

If the cost of gaining information, c, is non-negligible, then the equilibrium frequencies depend on this cost and on α, the stochastic nature of the environment, as illustrated in Fig. 3.

### Evolutionary stability

**Fig. 4.**
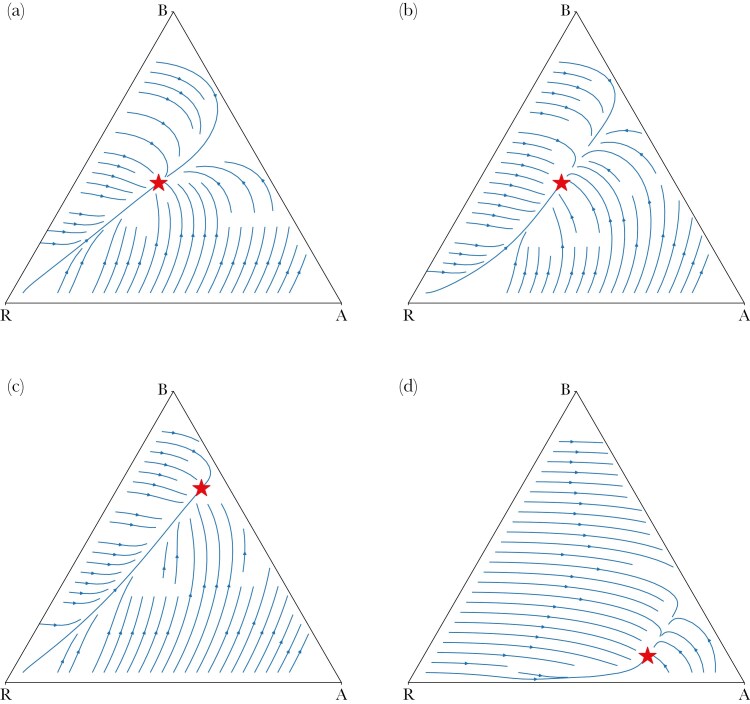
Phase diagram of the replicator dynamics for c=0, under a variety of different parameter specifications. The only difference between figures a and b is that the stochasticity parameter α changes. This has no effect on the equilibrium itself but does affect somewhat the out of equilibrium dynamics. It can also be seen that not only is the unique equilibrium asymptotically stable, but also in fact a global attractor under the replicator dynamics: All solution paths eventually converge to the equilibrium, (a) α = 0.7, λ = 0.7, η = 3, (b) α = 0.4, λ = 0.7, η = 3, (c) α = 0.4, λ = 2, η = 3, (d) α = 0.1, λ = 0.1, η = 0.5.

In online Appendix 10 we show that the unique symmetric Nash equilibrium in this model is also an evolutionarily stable strategy, in the sense of Palm (1984) , see also Broom et al. (1997), who have extended the definition of an ESS of Maynard Smith and Price (1973) to symmetric n-player games. Note that behind the notion of an ESS (and also behind the replicator and other dynamics) is the assumption that there is an infinite population of individuals, from which over and over again (always new—stochastically independently) n individuals are randomly drawn to play the game. Evolutionary forces are at work in this infinite population. We know generally, see eg Nachbar (1990), that Nash equilibria are the only candidates for asymptotically stable rest points under most deterministic behavioral adjustment (or evolutionary) dynamics, with the replicator dynamics of Taylor and Jonker (1978) the first and most prominent example. In online Appendix 11 we show that the games we here study are stable games in the sense of Hofbauer and Sandholm (2009), with the implication that the unique symmetric equilibrium of our model is asymptotically stable under many different dynamics that includes the replicator dynamics. In Fig. 4 we illustrate this finding by sketching the phase diagram of the replicator dynamics for different parameter configurations of our model.

### Consistency and correlation

Suppose that the n individuals play the same game given in our basic model over and over again for many periods of time. Suppose that, at every point in time, they play the unique symmetric equilibrium given earlier. An outside observer would note that when the amount of food available at B is high (X=η) more individuals are to be found at source B than when it is low (X=λ). They would also observe that the food share each individual receives is the same regardless of which source the individuals go to. The outside observer would conclude that some individuals must be responsive to the stochastic food availability at source B. However, there are, so far, no incentives for individuals to choose the same strategy across time and context.

Recall the two interpretations we can give a mixed strategy equilibrium, as we identified in this model. Suppose that we interpret it as the various individuals involved actually randomizing between the three pure strategies. In that case, the outside observer would note that the identity of responsive individuals varies over time. This, however, is inconsistent with empirical findings (of consistency and correlation as highlighted in the introduction). Alternatively we can interpret the mixed strategy equilibrium as there being a large population of individuals, with certain fractions of these individuals playing the various pure strategies. In this latter case, it is more plausible that if one individual is responsive at one point in time it is also responsive at another point in time. Note, however, that all individuals only have a very weak incentive to stay with their pure strategy choice, as (in any mixed equilibrium) all pure strategies provide the same expected benefit (food share in our model). In principle, the various individuals could switch pure strategies around, as long as the aggregate frequencies remain the same.

A slight change to the basic model, however, provides essentially the same prediction as the basic model, but now with every individual having a strict preference to play a pure strategy with frequencies very close to the mixed strategy equilibrium of the basic model.

This modification is based on the idea of *purification* of Harsanyi (1973), which is very similar to the idea of *threshold decisions* as provided in McNamara and Houston (2005). The idea is that individuals differ a little bit in terms of their personal preferences and actually make a pure strategy choice that is, however, dependent on their own personal preferences that only they themselves know. This idea is also present in the discussion of the stability of randomization Maynard Smith (1988), who first argues that randomization can be stable even in finite populations, but then offers the interpretation of randomized strategies as threshold strategies based on some individual personal characteristic, and gives the examples of age or size. As a consequence, while the equilibrium looks mixed to other individuals, each individual actually plays a pure strategy.

We adapt the model by replacing the payoff function u of the original model with a slightly perturbed payoff function $v_{\theta }$ that is essentially equal to u plus a small idiosyncratic (individual-specific) preference or perturbation term:


$$\begin{array}{l} \begin{array}{lllllllllllllllllllll} v_{\theta }\left(A,\sigma \right) & = & u\left(A,\sigma \right) & + & \theta_{A}\;v_{\theta }\left(B,\sigma \right) & = & u\left(B,\sigma \right) & + & \theta_{B}\;v_{\theta }\left(R,\sigma \right) & = & u\left(R,\sigma \right) & + & \alpha \theta_{B}+\left(1-\alpha \right)\theta_{A}+\theta_{R}\;v_{\theta }\left(C,\sigma \right) & = & u\left(C,\sigma \right) & + & \alpha \theta_{A}+\left(1-\alpha \right)\theta_{B}+\theta_{R},\; & & & & \; \end{array} \end{array}$$

where the vector $\theta =\left(\theta_{A},\theta_{B},\theta_{R}\right)$ is i.i.d. drawn from some arbitrary full support continuous joint distribution F (with density f) over $\;\Theta \;={\left[-\epsilon ,\epsilon \right]}^{3}$, for a small ϵ>0 (the assumption of independent idiosyncratic preference perturbations is not necessarily innocent. See eg Leimar et al. (2004) for an analysis of such cases in the context of environmental sex determination). It is assumed that an individual’s realized θ is that individual’s private information, which means that only this individual knows it; it is unknown to other individuals. We have deliberately chosen the same preference perturbation $\theta_{R}$ for pure strategies C and R, as it seems more natural to have an idiosyncratic perturbation of the cost of being responsive rather than for how one is responsive. However, it does not matter what we assume for pure strategy C as long as the payoff perturbation is small, as pure strategy C provides a strictly lower payoff than the other three strategies in the equilibrium given earlier, and small payoff perturbations cannot change that.

Harsanyi (1973) has shown that almost any equilibrium of a complete information game, such as our basic game, is such that any nearby incomplete information game, with payoff perturbations given by the joint distribution F, has a nearby equilibrium and that this nearby equilibrium is essentially in pure strategies. In such a nearby equilibrium there is a parameter region for θ∈ Θ  for which an individual strictly prefers to play A, another region for which an individual strictly prefers to play B, and a final region in which an individual strictly prefers to play R. The set of θ’s for which an individual is indifferent between two or three of the three strategies has measure zero (it is statistically impossible). For more details see online Appendix 8. Finally, such purified equilibria can also shown to be dynamically stable under a suitably defined behavioral adjustment dynamics as in Ely and Sandholm (2005), see Sandholm (2007) and also online Appendix 12 for a simulation of a (stochastic) process of threshold evolution.

In other words, the result of Harsanyi (1973) implies that each individual uses a pure strategy, which they strictly prefer given their own private preference, but the frequency of each strategy at the population level is essentially the same as without the preference perturbation.

Suppose now that the n individuals play the resulting equilibrium of the same slightly perturbed game repeatedly over many time periods. It is then a question of whether the perturbation parameters θ remain the same for each individual over time or not. If they do, it will be the same individuals who always go to food source A, the same individuals who always go to food source B, and the same who are responsive.

As an example of why the perturbed model may be appropriate for our purposes, consider birds who every day have to decide to go to food source A or B from their nesting place. Then the location of their nesting place gives rise to their θ. An approach could be that $\theta_{A}$ and $\theta_{B}$ are proportional to the distance that the bird’s nest is from the two food sources, respectively, while $\theta_{R}$ could be more of a personal characteristic of the bird, measuring how much/less cognitively able this bird is relative to other birds.

This model is then also flexible enough to generate a strong consistency over time and a weaker, but some, consistency across contexts, depending on how these consistencies are interpreted (in such a way, however, that the overall frequencies of choices remain close to the original equilibrium frequencies). Consider the bird example again. One could imagine that $\theta_{R}$ is an individual bird’s specific parameter that does not change over time nor across contexts. On the other hand, the parameters $\theta_{A}$ and $\theta_{B}$ might be constant for one season, but could be different in another season, when the bird’s nest location (or the location of the food sources) changes.

Equilibrium purification could even be obtained by introducing a payoff-irrelevant personal and privately know characteristic, such as an individual’s prior experiences in life, with individuals playing different pure strategies depending on their personal prior experiences. This means that, as pointed out eg in Wolf et al. (2007), Wolf and Weissing (2010) and Dingemanse and Wolf (2010), the purification threshold could also be based on an individual’s life history.

In other words, the phenotypic variation in individuals’ behavior, which is induced by the preference perturbations $\theta_{R}$, $\theta_{A}$, and $\theta_{B}$ could be genetic, environmentally induced, or even random. In the above example of birds, we have argued that $\theta_{R}$ could be the bird’s innate, ie genetic, predisposition to be responsive, perhaps based on the bird’s genetic cognitive ability. On the other hand, parameters $\theta_{A}$ and $\theta_{B}$ could be environmentally induced. In the example, they are based on the distance of the food sources from a bird’s nesting site. Some of these parameters could also be generated almost randomly if an individual were to have “whims of the moment,” that is, its preference perturbation is sometimes higher and sometimes lower. There could be an evolutionary advantage of such preference variation in some cases. If individuals’ relevant internal states are almost random, they are likely not measurable for an outside observer. This could explain why the meta-study by Niemelä and Dingemanse (2018) finds only a weak link between individuals’ state and their choices: either some of the studies measured the “wrong” states or some of the states are simply not measurable. A combination of these components is also likely required if one were to try to calibrate the model to generate observed estimates of consistency (or repeatability) of behavior, such as the overall average estimate of repeatability of behavior of 0.37 given in the meta-analysis of Bell et al. (2009, p. 774), or to match the range of diverse species-specific estimates given there for each species separately.

### Imperfect private signals of food availability

In our basic model, individuals can learn the state of food availability at the food sources perfectly. In this section, we study how the results change if this learning is imperfect. To do so, we suppose that each individual receives a noisy signal about the level of food availability at food source B. Individuals i receive conditionally independent (and identically distributed) signals $s_{i}\in \left\{l,h\right\}$ such that $P(s_{i}=h|X=\eta )=P(s_{i}=l|X=\lambda )=1-\epsilon$, with $\epsilon <\frac{1}{2}$. In words, in the high state η the high signal h is more likely than the low signal l, and in the low state λ the low signal l is more likely than the high signal h. The signal is, thus, informative about the true state.

All the arguments made for the perfect signals of the food availability model (the basic model) go through (see Online Appendix 9). Ultimately, we obtain that, provided the level of noise ϵ is not too large, the game has a unique symmetric Nash equilibrium, denoted $\sigma_{\epsilon }$, that is also an ESS (and dynamically stable under a large class of evolutionary adjustment models). In the limiting case when the cost of gaining information c→0 the equilibrium is given by


$$\begin{array}{l} \left\{ \begin{array}{lllllll} \sigma_{\epsilon }\left(R\right) & = & \frac{\eta -\lambda }{\left(1-2\epsilon \right)\left(1+\eta \right)\left(1+\lambda \right)}\;\sigma_{\epsilon }\left(A\right) & = & \frac{1}{1+\eta }-\epsilon \frac{\eta -\lambda }{\left(1-2\epsilon \right)\left(1+\eta \right)\left(1+\lambda \right)}\;\sigma_{\epsilon }\left(B\right) & = & \frac{\lambda }{1+\lambda }-\epsilon \frac{\eta -\lambda }{\left(1-2\epsilon \right)\left(1+\eta \right)\left(1+\lambda \right)}. \end{array}\right. \end{array}$$

**Fig. 5.**
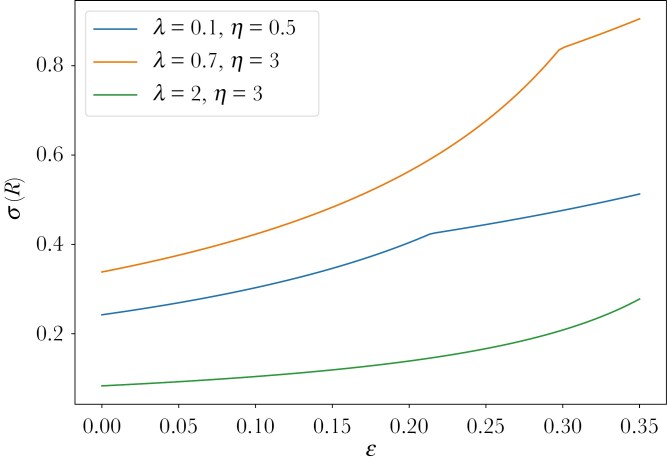
The proportion of responsive individuals increases with the level of noise in the information. The kink in the graph is at the level of noise ϵ at which one of the two equilibrium frequencies $\sigma_{\epsilon }\left(A\right)$ or $\sigma_{\epsilon }\left(B\right)$ become zero.


There are two interesting implications of this model, both seen also in Fig. 5. First, for all parameter values λ,η, the frequency of responsive individuals increases with the level of noise ϵ in the information. The noisier the signal the more individuals are responsive to the possibly erroneous information about the stochastic food source. The intuition behind this is, that because of the possible error, more individuals have to respond, because some will get the wrong signal and, thus, respond incorrectly. This would lead to not enough individuals responding appropriately. Second, the impact that an increase of the level of noise ϵ has on the frequency of responsive individuals is higher the higher the frequency of responsive individuals is without noise. This can be seen in Fig. 5, and also formally, if we write

$$\begin{array}{l} \sigma_{\epsilon }\left(R\right)=\frac{1}{1-2\epsilon }\sigma_{0}\left(R\right), \end{array}$$

and, taking the derivative with respect to ϵ obtain


$$\begin{array}{l} \frac{d}{d\epsilon }\sigma_{\epsilon }\left(R\right)=\frac{2}{{\left(1-2\epsilon \right)}^{2}}\sigma_{0}\left(R\right). \end{array}$$

Interestingly, these results remain valid even when considering a strictly positive cost of gaining information.

### General distributions of food source availability

Recall that in the basic model food source B is assumed to follow a binary distribution (ie with only two possible levels of available food at that source). In this section we consider an arbitrary distribution for the food availability at food source B.

Let X, the available quantity of food at food source B, be distributed according to some distribution with cdf F with everywhere positive density f on the interval $\chi =\left[x_{L},x_{H}\right]$ with $0\leq x_{L}<x_{H}\leq \infty$. To make the analysis tractable we simplify the model in two ways. First, we set the cost of being responsive, c, to zero. Second, we assume that all individuals learn the value of X, and allow individuals to only use monotone strategies: An individual’s strategy can be described by a cutoff value y∈χ such that the individual goes to food source A if and only if x<y. Otherwise the individual goes to food source B. This implies that the strategy space is identical to χ and the set of mixed strategies is the set  Δ(χ) of all probability distributions over χ. A fully mixed symmetric Nash equilibrium strategy, which can be described by a cdf G on χ must satisfy that any individual is indifferent between using any cutoff y∈χ.

We then get the following result. In the model of this section, for any n, there is a unique completely mixed symmetric equilibrium. In the limit as n tends to infinity, the equilibrium probability that an individual uses cutoff responsiveness y is given by the cdf $G\left(y\right)=\frac{y}{1+y}$, with $G\left(x_{L}\right)=\frac{x_{L}}{1+x_{L}}$ the probability of an individual always going to food source B, $1-G\left(x_{H}\right)=\frac{1}{1+x_{H}}$ the probability of an individual always going to food source A, and $G\left(y\right)-G\left(x\right)=\frac{y-x}{\left(1+x\right)\left(1+y\right)}$ the probability that an individual adopts a degree of responsiveness in the interval [x,y]. The distribution with cdf $G\left(y\right)=\frac{y}{1+y}$ is the distribution of a random variable Y such that its reciprocal (or inverse) $\frac{1}{Y}$ has exactly the same distribution. One could call G the inversion invariant distribution.

This finding is consistent with those in the basic model. For example, the strategy called B in the previous model is similar to choosing the cutoff $x_{L}$ (since $x_{L}$ is the minimum possible value of the stochastic source). The equilibrium frequency of this strategy in the basic model is given by $\frac{\lambda }{1+\lambda }$, which is equivalent to $\frac{x_{L}}{1+x_{L}}$, as $x_{L}$ is the smallest possible value of the stochastic source. This generalization to the basic model also delivers a new insight. If there is a wide range of possible levels of food availability at the stochastic food source, then in equilibrium there is a continuum of degrees of responsiveness to environmental stimuli. See eg Lionetti et al. (2018) for empirical support for this finding.

## Discussion

We built a stylized game-theoretic model of foraging behavior in a stochastic environment. For every parameter specification within certain bounds, this model has a unique symmetric Nash equilibrium, that is also the unique ESS and asymptotically stable under a variety of evolutionary dynamics. This equilibrium has the three key features identified in the literature of *coexistence* of differing degrees of environmental responsiveness, *consistency* of individual responsiveness over time, and *correlation* of individual responsiveness across contexts.

By explicitly studying the phenomenon of heterogeneous responsiveness to environmental stimuli in a foraging setting, we are able to identify the push towards the ideal free distribution of Fretwell and Lucas (1969), satisfied in the equilibrium of our game, as a possible driving force of this heterogeneity. See also Křivan et al. (2008) for a game-theoretic treatment.

In our model, when there is no cost associated with responsiveness, the payoff distributions at equilibrium are identical, regardless of the strategies employed. This suggests that risk sensitivity does not play a role in determining the use of different strategies. Typically, risk-sensitive foraging refers to situations where choices are influenced by the variability in returns as well as the average returns from various options. For a recent review of risk-sensitive foraging, see Houston and Rosenström (2024).

However, when there is a small cost associated with responsiveness, we observe a pattern of undermatching for high-resource sources and overmatching for low-resource sources. This behavior has been documented by Kennedy and Gray (1993), Abrahams (1986). Additionally, DiNuzzo and Griffen (2020) examined the impact of animal personality on the ideal free distribution, suggesting that individual personality traits could be a contributing factor to this observed undermatching pattern.

We derive explicit analytical expressions for the equilibrium frequencies of responsive and non-responsive behavior, at least when the cost of gaining information (needed to respond to environmental stimuli) is negligible or at least relatively small. This allows us to study how the equilibrium frequencies change when some of the model parameters change. For instance, we find that, at least when the cost of gaining information is negligible, the exact stochastic nature of the environment does not affect the equilibrium. This finding suggests that changes in the stochastic environmental process would at least not be so disruptive as to push behavior out of equilibrium. Put differently, equilibrium strategies are already complex enough to allow for automatic adaptation to such changes in the stochastic environmental process. In the remainder of this section we discuss some of the limitations of our approach.

### Cost of gaining information

We have mostly explored the case of zero, and by a continuity argument, also of small cost of gaining information. For larger cost of gaining information, generally, equilibrium behavior will depend on the stochastic nature of the environment, see, for instance, Fig. 3, and the equilibrium will not satisfy the ideal free distribution. One could possibly consider the cost of gaining information also as a cost of having a sufficient degree of cognitive ability. For most species, it is not unreasonable to assume a relatively small cost of cognitive ability, see eg (van Buskirk and Steiner 2009; Auld et al. 2010; Murren et al. 2015; Hendry 2016). Relatedly, the cost of plasticity, as defined, for instance, in DeWitt et al. (1998), see also Relyea (2002) is typically small; see, for instance Hendry (2016), the review by Murren et al. (2015), and the meta-analysis by Van Buskirk and Steiner (2009).

### Noisy information

Another insight that we can derive from an extension of our model is that the higher the noise in the environmental stimuli the more responsive individuals become in equilibrium. This is under the assumption of individuals receiving private and stochastically independent noisy information about the state of the environment. We have not explored the case of correlated information, such as all individuals receiving the same public information. In such a setting, the ideal free distribution would at best hold in expectation, and there would be a positive variance of food share availability at the random source.

### Social Information

Another, empirically relevant, informational setting, that we here abstracted away from, is one where not all individuals receive the same quality of information (perhaps not all are equally close to the source of information). One would then expect individuals to infer additional information about the state of the environment from other individuals’ behavior. If, for instance, there are many birds flying out to a specific point at sea, another bird might follow them based on the idea that there is information in that behavior. This will certainly be the case for socializing birds, which display behavior of forming flocks and swarms. Such behavior would add another layer of complexity to the game. For a literature review on social information use in a foraging context see Kohles et al. (2022). According to the “information-sharing theory,” as in Clark and Mangel (1984), it has been observed for different species that animals can both search for food on their own or join others who have found food, see eg Giraldeau and Beauchamp (1999). Models, in which individuals choose between the two strategies “discoverers” or “copiers” are referred to as “producer-scrounger games” (Giraldeau and Beauchamp 1999; Bhattacharya and Vicsek 2014).The interest in using social information is notably to avoid paying a cost to acquire private information (Webster and Laland 2008). The choice between producer and scrounger is, to some extent, persistent in time (Morand-Ferron et al. 2011). The connection between *responsiveness* and the producer-scrounger game could be made through the concepts of *personality*. Indeed, the main trait of personality is the axis boldness-shyness, and it has been observed that shyness increases the probability to scrounge (Kurvers et al. 2010).

### The number of food sources

Our model only has two food sources. This keeps the analysis mathematically tractable, but at the cost of a possible oversimplification. Given our results, however, one would conjecture that in any (evolutionary stable) equilibrium of a game with multiple food sources, the ideal free distribution holds, at least when costs of gaining information are negligible: all food sources would have equal food shares, and this would be true for all states. This would imply that equilibrium behavior would not depend on the exact stochastic nature of the environment. This would also imply that any (evolutionary stable) equilibria would again satisfy coexistence, and for slightly perturbed models, consistency and correlation. One would, however, not necessarily expect a unique equilibrium and it would be harder to characterize these explicitly.

### Generalizing

We focused here on foraging choice as a concrete setting in which one would expect the coexistence, consistence and correlation of different responsiveness to external stimuli. However, our results could possibly be generalized to different context where there is a resource to share among individuals, with a resource distributing at different point, and some of them stochastic. Such contexts are social interactions, mating behaviour, division of labor (Dall et al. 2012) space-use (Ceia and Ramos 2015; Spiegel et al. 2017), or niche specialization (Bolnick et al. 2003; Dall et al. 2012; Montiglio et al. 2013). Those last studies show similar concepts (state dependence, frequency dependence, social awareness, environmental heterogeneity) applied to niche specialization. In particular, increasing evidence show a link between specialization and personality. It is hypothesized that personality implies specialization (Toscano et al. 2016; Harris et al. 2020) or the other way around (Bergmüller and Taborsky 2010; Montiglio et al. 2013). Similar to our model, Leimar et al. (2022). Similar to our model, Leimar et al. (2022) used frequency-dependent competition for resources to explain specialization. For a review of individual foraging specialization see Sheppard et al. (2021).

## Supplementary Material

araf023_suppl_Supplementary_Appendix

## Data accessibility

This study does not involve any empirical data. All results are derived from theoretical modeling and mathematical analysis.
