# Supplementary material for: Heterogeneous responsiveness to environmental stimuli
Source: Behav Ecol. 2025 Aug 16;36(4):araf023. doi: 10.1093/beheco/araf023 (PMC12357134; doi:10.1093/beheco/araf023)
Supplement: araf023_suppl_Supplementary_Appendix [file araf023_suppl_supplementary_appendix.pdf]

A Equilibrium

Definition of payoffs for any number of players Denote by N_s the random variable that is the number of opponents who end up choosing pure strategy $s \in S$ (given the probability of choosing s is $\sigma(s)$). The tuple (N_A, N_B, N_R, N_C) is then multinomially distributed with the probability vector $(\sigma(A), \sigma(B), \sigma(R), \sigma(C))$.

Given σ, let $R_{A\lambda}$ denote the random variable that is the food share available at food source A in state λ. Let food shares $R_{A\eta}$, $R_{B\lambda}$, $R_{B\eta}$, be defined analogously. These are given by

$$\begin{array}{rclrcl} R_{A\lambda} & = & \frac{n}{N_A+N_R+1} & R_{A\eta} & = & \frac{n}{N_A+N_C+1} \\ R_{B\lambda} & = & \frac{\lambda \cdot n}{N_B+N_C+1} & R_{B\eta} & = & \frac{\eta \cdot n}{N_B+N_R+1}. \end{array}$$

We can, then, express an individual's expected payoffs from choosing pure strategy $s \in S$, when all others use mixed strategy σ, as follows.

$$\begin{array}{rcl} u(A,\sigma) & = & \alpha \mathbf{E}\left[R_{A\eta}\right] + (1-\alpha)\mathbf{E}\left[R_{A\lambda}\right] \\ u(B,\sigma) & = & \alpha \mathbf{E}\left[R_{B\eta}\right] + (1-\alpha)\mathbf{E}\left[R_{B\lambda}\right] \\ u(R,\sigma) & = & \alpha \mathbf{E}\left[R_{B\eta}\right] + (1-\alpha)\mathbf{E}\left[R_{A\lambda}\right] - c \\ u(C,\sigma) & = & \alpha \mathbf{E}\left[R_{A\eta}\right] + (1-\alpha)\mathbf{E}\left[R_{B\lambda}\right] - c, \end{array} \tag{1}$$

where $\mathbf{E}$ denotes the expectation with respect to the randomness created by mixed strategy σ.[1] We extend an individual's payoff function to mixed strategies by taking expectations:

$$u(\sigma', \sigma) = \sum_{s \in S} u(s, \sigma)\sigma'(s).$$

Strategy $\sigma \in \Delta(S)$ is a *symmetric equilibrium* [Nash, 1950] if $u(\sigma, \sigma) \geq u(\sigma', \sigma)$ for all $\sigma' \in \Delta(S)$.

Deriving the unique equilibrium We first show that pure strategies A, B, and R, cannot be equilibria for any $c > 0$ and for any n given the assumption that $\frac{1}{n} < \lambda < \eta < n$. Suppose everyone plays A. Then everyone gets a payoff of $\frac{1}{n}$. An individual deviating to A would at least get λ and since $\lambda > \frac{1}{n}$ this is a profitable deviation. Suppose everyone plays B. Then everyone gets at most $\eta < n$ (if $X = \eta$ and less if $X = \lambda$). Deviating to B would yield a payoff of n. Suppose, finally, that everyone plays R. Then

[1] For instance, $\mathbf{E}\left[R_{A\lambda}\right] = \sum_{k_A=0}^{n-1} \sum_{k_R=0}^{n-1-k_A} \frac{n}{k_A+k_R+1} \frac{(n-1)!}{k_A! k_R! (n-k_A-k_R)!} \sigma(A)^{k_A} \sigma(R)^{k_R} (1-\sigma(A)-\sigma(R))^{n-k_A-k_R}$.

everyone gets a payoff of either 1 (if $X = \lambda$ and everyone goes to food source A) or η (if $X = \eta$ and everyone goes to food source B), minus c, and then a deviation to B gives a higher payoff of $\lambda n > 1$ when $X = \lambda$ (this individual is alone at food source B) and a payoff of $n > \eta$ if $X = \eta$ (this individual is alone at food source A), and thus, a strictly higher expected payoff.

Next we show that there cannot be a symmetric Nash equilibrium that attaches non-zero probability to both pure strategies R and C. If this were the case, then necessarily $u(C,\sigma) = u(R,\sigma)$ and, by the fact that $u(A,\sigma) + u(B,\sigma) = u(R,\sigma) + u(C,\sigma) - 2c$ for all $\sigma \in \Delta(S)$, either pure strategy A or B (or both) provide a strictly higher payoff than both R and C.

There cannot be a symmetric Nash equilibrium in which strategy C is used with non-zero probability. To see this, we show that whenever C is used R must be used with non-zero probability. As we just showed that there cannot be an equilibrium with both C and R, this then proves that there is no equilibrium in which C is used.

We prove this by contradiction. Suppose, that $\sigma(R) = 0$ and $\sigma(C) > 0$ for an equilibrium $\sigma \in \Delta(S)$. We will show that, in this case, either strategy A or B will provide a strictly better payoff than C does. Noting that $N_R = 0$, we can write the expected payoffs as

$$\begin{array}{rcl} u(A,\sigma) &=& \alpha \mathbf{E}\left[\frac{n}{N_A+N_C+1}\right] + (1-\alpha)\mathbf{E}\left[\frac{n}{N_A+1}\right] \\ u(B,\sigma) &=& \alpha \mathbf{E}\left[\frac{\eta\cdot n}{N_B+1}\right] + (1-\alpha)\mathbf{E}\left[\frac{\lambda\cdot n}{N_B+N_C+1}\right] \\ u(C,\sigma) &=& \alpha \mathbf{E}\left[\frac{n}{N_A+N_C+1}\right] + (1-\alpha)\mathbf{E}\left[\frac{\lambda\cdot n}{N_B+N_C+1}\right] - c. \end{array}$$

As σ is an equilibrium we must have $u(A,\sigma), u(B,\sigma) \le u(C,\sigma)$. This implies that

$$\mathbf{E}\left[\frac{\lambda}{N_B+N_C+1}\right] \ge \mathbf{E}\left[\frac{1}{N_A+1}\right]$$

$$\mathbf{E}\left[\frac{1}{N_A+N_C+1}\right] \ge \mathbf{E}\left[\frac{\eta}{N_B+1}\right]$$

But, since we have $\mathbf{E}[N_C] > 0$, we have

$$\mathbf{E}\left[\frac{1}{N_A+1}\right] > \mathbf{E}\left[\frac{1}{N_A+N_C+1}\right] \text{ and } \mathbf{E}\left[\frac{\lambda}{N_B+1}\right] > \mathbf{E}\left[\frac{\lambda}{N_B+N_C+1}\right],$$

which, in combination with the previous two inequalities yields

$$\begin{array}{rcl} \mathbf{E}\left[\frac{\lambda}{N_B+1}\right] > \mathbf{E}\left[\frac{\lambda}{N_B+N_C+1}\right] &\ge& \mathbf{E}\left[\frac{1}{N_A+1}\right] \\ > \mathbf{E}\left[\frac{1}{N_A+N_C+1}\right] &\ge& \mathbf{E}\left[\frac{\eta}{N_B+1}\right], \end{array}$$

and, thus,

$$\mathbf{E}\left[\frac{\lambda}{N_B+1}\right] > \mathbf{E}\left[\frac{\eta}{N_B+1}\right],$$

which, finally, contradicts our assumption that $\eta > \lambda$.

Next we show that, given the assumption that $\frac{1}{n} < \lambda < \eta < n$, there cannot be any mixed strategy equilibrium with only two pure strategies in its support. We have already shown that there cannot be an equilibrium with C in its support. Suppose there is an equilibrium σ that has a support of $\{A, R\}$. Then $u(A, \sigma) = u(R, \sigma)$. However, $u(B, \sigma) > u(R, \sigma)$, as B provides the same payoff as R when $X = \eta$ and obtains λn when $X = \lambda$ (as everyone else is at source A), while R obtains $1 < \lambda n$. Suppose there is an equilibrium σ that has a support of $\{B, R\}$. Then $u(B, \sigma) = u(R, \sigma)$. However, $u(A, \sigma) > u(R, \sigma)$, as A provides the same payoff as R when $X = \lambda$ and obtains n when $X = \eta$ (as everyone else is at source B), while R obtains $\eta < n$.

Suppose there is an equilibrium σ that has a support of $\{A, B\}$. Then $u(A, \sigma) = u(B, \sigma)$. As $\eta > \lambda$, then for sufficiently small costs c, R provides a higher payoff than both A and B. In expectation both food sources A and B have the same food share, but R provides a higher payoff by going to B if and only if $X = \eta$.

This, finally, leaves as the only possible equilibria those with support given by $\{A, B, R\}$. Let $r_{yx} = \mathbf{E}[R_{yx}]$ denote the expected food share at source $y \in \{A, B\}$ in state $x \in \{\lambda, \eta\}$. Consider $r_{A\lambda}$. All other expected food shares are calculated analogously. We can write $r_{A\lambda} = n\mathbf{E}[\frac{1}{Y+1}]$, where $Y = N_A + N_R$, and Y, thus, binomially distributed with $n-1$ tries and success probability $p = \sigma(A) + \sigma(R)$. The first negative moment of such a binomial distribution, $\mathbf{E}[\frac{1}{Y+1}]$, is provided in [Chao and Strawderman, 1972, Equation 3.4] and given by $\frac{1-(1-p)^n}{np}$.

We, thus, obtain

$$\begin{aligned} r_{A\lambda} &= \tfrac{1-(1-\sigma(A)-\sigma(R))^n}{\sigma(A)+\sigma(R)}, \\ &\text{and analogously} \\ r_{A\eta} &= \tfrac{1-(1-\sigma(A)-\sigma(C))^n}{\sigma(A)+\sigma(C)} \\ r_{B\lambda} &= \lambda \cdot \tfrac{1-(1-\sigma(B)-\sigma(C))^n}{\sigma(B)+\sigma(C)} \\ r_{B\eta} &= \eta \cdot \tfrac{1-(1-\sigma(B)-\sigma(R))^n}{\sigma(B)+\sigma(R)}. \end{aligned} \tag{2}$$

We need to show that there is a unique equilibrium with support $\{A, B, R\}$. To be indifferent between A and R and B and R requires that

$$r_{A\eta} = r_{B\eta} - \frac{c}{\alpha} \text{ and } r_{B\lambda} = r_{A\lambda} - \frac{c}{1-\alpha}.$$

Plugging in the above expressions, and noting that $\sigma(C) = 0$, these two equations lead to

$$\frac{1-(1-\sigma(A))^n}{\sigma(A)} = \eta\frac{1-\sigma(A)^n}{1-\sigma(A)} - \frac{c}{\alpha}, \tag{3}$$

and

$$\lambda\frac{1-(1-\sigma(B))^n}{\sigma(B)} = \frac{1-\sigma(B)^n}{1-\sigma(B)} - \frac{c}{1-\alpha}, \tag{4}$$

respectively.

In general, we cannot provide an analytic solution to this system of equations. If, however, we consider the limit of the number of individuals going to infinity, $n \to \infty$, the system of equations simplifies somewhat to

$$\frac{1}{\sigma(A)} = \eta\frac{1}{1-\sigma(A)} - \frac{c}{\alpha}, \tag{5}$$

and

$$\lambda\frac{1}{\sigma(B)} = \frac{1}{1-\sigma(B)} - \frac{c}{1-\alpha}, \tag{6}$$

respectively.

These are quadratic equations that have the following unique closed form solution (in the relevant range between 0 and 1).

$$\begin{aligned}
\sigma(A) &= \tfrac{1}{2c}\left[\sqrt{(\alpha(1+\eta)-c)^2+4\alpha c} - (\alpha(1+\eta)-c)\right] \\
\sigma(B) &= \tfrac{1}{2c}\left[\sqrt{((1-\alpha)(1+\lambda)-c)^2+4(1-\alpha)\lambda c} - ((1-\alpha)(1+\lambda)-c)\right] \\
\sigma(R) &= \tfrac{1}{2c}\Big[1+\alpha\eta+(1-\alpha)\lambda - \sqrt{(\alpha(1+\eta)-c)^2+4\alpha c)} \\
&\quad - \sqrt{((1-\alpha)(1+\lambda)-c)^2+4\lambda(1-\alpha)c)}\Big]
\end{aligned}$$

If, moreover, we consider the limit case of costs of gaining information going to zero, $c \to 0$, the system of equations further simplifies to

$$\frac{1}{\sigma(A)} = \eta\frac{1}{1-\sigma(A)} \text{ and } \lambda\frac{1}{\sigma(B)} = \frac{1}{1-\sigma(B)}, \text{ respectively.}$$

The unique solution is then $\sigma(A) = \frac{1}{1+\eta}$ and $\sigma(B) = \frac{\lambda}{1+\lambda}$. This is the only candidate for an equilibrium of this form. As $\sigma(R) = 1 - \sigma(A) - \sigma(B)$ we obtain $\sigma(R) = \frac{\eta-\lambda}{(1+\eta)(1+\lambda)}$. Note, finally, that $\sigma(A), \sigma(B), \sigma(R) \geq 0$, and, thus σ is indeed the unique (mixed strategy) equilibrium.

B General distributions

Let $u(A, G, x)$ and $u(B, G, x)$ denote the expected payoff to an individual who goes to food source A and B, respectively, when all others use mixed strategy G and when the realized state of food availability at food source B is given by x. As $G(x) = P(y \leq x)$ is the probability that a random other individual goes to food source B, using again [Chao and Strawderman, 1972, Equation 3.4], we have

$$u(A, G, x) = \frac{1 - G(x)^n}{1 - G(x)}$$

and

$$u(B, G, x) = x\frac{1 - (1 - G(x))^n}{G(x)}.$$

The expected payoff for an individual using cut-off $y \in \chi$, when all others use mixed strategy G, is then given by

$$u(y, G) = \int_{x=x_L}^{y} \frac{1 - G(x)^n}{1 - G(x)} f(x)dx + \int_{x=y}^{x_H} x\frac{1 - (1 - G(x))^n}{G(x)} f(x)dx.$$

For a completely mixed strategy G to be a symmetric Nash equilibrium we need that $u(y, G)$ is constant for all $y \in \chi$. If $u(y, G)$ is a constant function in y then its derivative with respect to y must be constant as well (and equal to zero). This implies that

$$\frac{1 - G(y)^n}{1 - G(y)} f(y) - y\frac{1 - (1 - G(y))^n}{G(y)} f(y) = 0,$$

for all $y \in \chi$. Note that $f(y)$ cancels out and the resulting equilibrium distribution G does again not depend on the stochasticity of the food source, and we obtain

$$\frac{1 - G(y)^n}{1 - G(y)} = y\frac{1 - (1 - G(y))^n}{G(y)}.$$

Note that the left hand side of this equation is increasing in $G(y)$ and the right hand side is decreasing in $G(y)$. Note also that the left hand side is strictly less than the right hand side when both are evaluated at $G(y) = 0$ and the inequality is reversed when both are evaluated at $G(y) = 1$. Thus, for any $y \in \chi$, this equation has a unique solution $G(y)$. Note that this solution $G(y)$ must necessarily be higher for higher y. Note also that, necessarily, $G(0) = 0$ and $\lim_{y\to\infty} G(y) = 1$. G is, thus, a cumulative distribution function.

For finite n this solution is not easily expressed analytically. In the limit as $n \to \infty$ we obtain

$$\frac{1}{1-G(y)} - y\frac{1}{G(y)} = 0,$$

for all $y \in \chi$, and, thus, $G(y) = \frac{y}{1+y}$ in this limiting case.

C Harsanyi Purification

In the perturbed game, a strategy is a function $\rho : \Theta \to \Delta(S)$, where ρ attaches to each possible preference type θ a probability distribution over S. Every such strategy ρ induces a strategy $\sigma^\rho \in \Delta(S)$ by $\sigma^\rho(s) = \int_\Theta \rho(\theta)(s) f(\theta) d\theta$ for all $s \in S$, where $\rho(\theta)(s)$ is the probability that $\rho(\theta)$ attaches to s. An equilibrium strategy ρ is then such that $\rho(\theta)(A) = 1$ if

$$\begin{array}{rcl} u(A,\sigma^\rho) - u(B,\sigma^\rho) & > & \theta_B - \theta_A, \text{ and} \\ u(A,\sigma^\rho) - u(R,\sigma^\rho) & > & \alpha(\theta_B - \theta_A) + \theta_R; \end{array}$$

$\rho(\theta)(B) = 1$ if

$$\begin{array}{rcl} u(A,\sigma^\rho) - u(B,\sigma^\rho) & < & \theta_B - \theta_A, \text{ and} \\ u(B,\sigma^\rho) - u(R,\sigma^\rho) & > & (1-\alpha)(\theta_A - \theta_B) + \theta_R; \end{array}$$

and $\rho(\theta)(R) = 1$ if

$$\begin{array}{rcl} u(A,\sigma^\rho) - u(R,\sigma^\rho) & < & \alpha(\theta_A - \theta_B) + \theta_R, \text{ and} \\ u(B,\sigma^\rho) - u(R,\sigma^\rho) & < & (1-\alpha)(\theta_A - \theta_B) + \theta_R. \end{array}$$

D Imperfect signals

In this case, (random) food shares are given by

$$\begin{array}{rcl} R_{A\lambda} & = & \frac{n}{N_{A\lambda}+1} \\ R_{A\eta} & = & \frac{n}{N_{A\eta}+1} \\ R_{B\lambda} & = & \frac{\lambda \cdot n}{N_{B\lambda}+1} \\ R_{B\eta} & = & \frac{\eta \cdot n}{N_{B\eta}+1}, \end{array}$$

where, given strategy σ (used by all opponents), $N_{A\lambda}$ follows a binomial distribution with $n-1$ trials and success probability $\sigma(A) + (1-\epsilon)\sigma(R) + \epsilon\sigma(C)$. Similarly, $N_{A\eta}, N_{B\lambda}, N_{B\eta}$ are also binomially distributed with $n-1$ trials and success probabilities $\sigma(A) + (1-\epsilon)\sigma(C) + \epsilon\sigma(R)$, $\sigma(B) + (1-\epsilon)\sigma(R) + \epsilon\sigma(C)$, and $\sigma(B) + (1-\epsilon)\sigma(C) + \epsilon\sigma(R)$, respectively.

We, thus, obtain

$$\begin{array}{rcl} r_{A\lambda} & = & \frac{1-(1-\sigma(A)-(1-\epsilon)\sigma(R)-\epsilon\sigma(C))^n}{\sigma(A)+(1-\epsilon)\sigma(R)+\epsilon\sigma(C)}, \\ & \text{and analogously} & \\ r_{A\eta} & = & \frac{1-(1-\sigma(A)-(1-\epsilon)\sigma(C)-\epsilon\sigma(R))^n}{\sigma(A)+(1-\epsilon)\sigma(C)+\epsilon\sigma(R)} \\ r_{B\lambda} & = & \lambda\cdot\frac{1-(1-\sigma(B)-(1-\epsilon)\sigma(C)-\epsilon\sigma(R))^n}{\sigma(B)+(1-\epsilon)\sigma(C)+\epsilon\sigma(R)} \\ r_{B\eta} & = & \eta\cdot\frac{1-(1-\sigma(B)-(1-\epsilon)\sigma(R)-\epsilon\sigma(C))^n}{\sigma(B)+(1-\epsilon)\sigma(R)+\epsilon\sigma(C)}. \end{array} \tag{7}$$

We need to show that there is a unique equilibrium with support $\{A, B, R\}$. To be indifferent between A and R requires that

$$r_{A\eta} = r_{B\eta} - \frac{c}{\alpha},$$

to be indifferent between B and R requires

$$r_{B\lambda} = r_{A\lambda} - \frac{c}{1-\alpha}.$$

Plugging in the above expressions, and noting that $\sigma(C) = 0$, these two equations lead to

$$\frac{1-(1-\sigma(A)-\epsilon\sigma(R))^n}{\sigma(A)+\epsilon\sigma(R)} = \eta\frac{1-(\sigma(A)+\epsilon\sigma(R))^n}{1-\sigma(A)-\epsilon\sigma(R)} - \frac{c}{\alpha}, \tag{8}$$

and

$$\lambda\frac{1-(1-\sigma(B)-\epsilon\sigma(R))^n}{\sigma(B)+\epsilon\sigma(R)} = \frac{1-(\sigma(B)+\epsilon\sigma(R))^n}{1-\sigma(B)-\epsilon\sigma(R)} - \frac{c}{1-\alpha}, \tag{9}$$

respectively. We are interested in the limit as $n \to \infty$ and $c \to 0$ and obtain

$$\frac{1}{\sigma(A)+\epsilon\sigma(R)} = \eta\frac{1}{1-\sigma(A)-\epsilon\sigma(R)},$$

and

$$\lambda\frac{1}{\sigma(B)+\epsilon\sigma(R)} = \frac{1}{1-\sigma(B)-\epsilon\sigma(R)},$$

respectively. Solving these two equations yields unique solutions $\sigma(A) + \epsilon\sigma(R) = \frac{1}{1+\eta}$ and $\sigma(B) + \epsilon\sigma(R) = \frac{\lambda}{1+\lambda}$. This is the only candidate for an equilibrium of this form. As $\sigma(R) = 1 - \sigma(A) - \sigma(B)$ we obtain $\sigma(R) = \frac{\eta-\lambda}{(1-2\epsilon)(1+\eta)(1+\lambda)}$ and then $\sigma(A) = \frac{1}{1+\eta} - \epsilon\frac{\eta-\lambda}{(1-2\epsilon)(1+\eta)(1+\lambda)}, \sigma(B) = \frac{\lambda}{1+\lambda} - \epsilon\frac{\eta-\lambda}{(1-2\epsilon)(1+\eta)(1+\lambda)}$. Note, finally, that $\sigma(A), \sigma(B), \sigma(R) \geq 0$, and, thus σ is indeed the unique (mixed strategy) equilibrium.

When ϵ gets large enough, one of the nonreactive strategies A or B can vanish. Then, the equilibrium changes. For example, if B vanishes. Then, the only equation is $\sigma(A) + \epsilon\sigma(R) = \frac{1}{1+\eta}$ with $\sigma(A) + \sigma(R) = 1$, which give the equilibrium $\sigma(R) = \frac{\eta}{(1-\epsilon)(1+\eta)}, \sigma(A) = \frac{1}{1+\eta} - \epsilon\frac{\eta}{(1-\epsilon)(1+\eta)}$

E Evolutionary Stable Strategy

We here show that the unique symmetric equilibrium of the basic game is evolutionarily stable as defined, for symmetric n player games, by Palm [1984], see also Broom et al. [1997]. By [Palm, 1984, Proposition 4] a sufficient condition for a (mixed) strategy σ of a symmetric n-player normal form game is an ESS is that

1. $u(\sigma, \sigma) \geq u(\sigma', \sigma)$ for all $\sigma' \in \Delta(S)$ and
2. if $u(\sigma', \sigma) = u(\sigma, \sigma)$ for some $\sigma' \neq \sigma$ then $u(\sigma', \sigma') < u(\sigma, \sigma')$.

The first condition states that only symmetric Nash equilibria can be an ESS. Thus, the strategy we identified as the unique symmetric Nash equilibrium of the game is the only candidate for an ESS. To show that it is indeed an ESS we need to show that it also satisfies the second condition.

For strictly positive costs pure strategy C yields a strictly lower payoff than the equilibrium payoff. However, all strategies $\sigma' \in \Delta(S)$ with $\sigma'(C) = 0$ satisfy $u(\sigma', \sigma) = u(\sigma, \sigma)$. We need to show that for all such $\sigma' \neq \sigma$ we have $u(\sigma, \sigma') - u(\sigma', \sigma') > 0$. As $\sigma'(C) = \sigma(C) = 0$. We have that,

$$\begin{array}{lr} u(\sigma, \sigma') - u(\sigma', \sigma') & = \\ (\sigma(A) - \sigma'(A)) \left(\alpha \frac{1-(1-\sigma'(A))^n}{\sigma'(A)} + (1-\alpha) \frac{1-\sigma'(B)^n}{1-\sigma'(B)} \right) & + \\ (\sigma(B) - \sigma'(B)) \left(\alpha\eta \frac{1-\sigma'(A)^n}{1-\sigma'(A)} + (1-\alpha)\lambda \frac{1-(1-\sigma'(B))^n}{\sigma'(B)} \right) & + \\ (\sigma(R) - \sigma'(R)) \left(\alpha\eta \frac{1-\sigma'(A)^n}{1-\sigma'(A)} + (1-\alpha) \frac{1-\sigma'(B)^n}{1-\sigma'(B)} - c \right). & \end{array}$$

As $\sigma(R) = 1 - \sigma(A) - \sigma(B)$ (and the same for σ'), this is equivalent to

$$\begin{array}{lr} u(\sigma, \sigma') - u(\sigma', \sigma') & = \\ \alpha \left(\sigma'(A) - \sigma(A)\right) \left(\frac{1-\sigma'(A)^n}{1-\sigma'(A)} \eta - \frac{1-(1-\sigma'(A))^n}{\sigma'(A)} - \frac{c}{\alpha} \right) & + \\ (1-\alpha) \left(\sigma'(B) - \sigma(B)\right) \left(\frac{1-\sigma'(B)^n}{1-\sigma'(B)} - \lambda \frac{1-(1-\sigma'(B))^n}{\sigma'(B)} - \frac{c}{1-\alpha} \right). & \end{array}$$

Note that last term in brackets in the first line is the equilibrium equation 5 (here for $\sigma'(A)$) and similarly the last term in brackets in the second line is the equilibrium equation 6 (here for $\sigma'(B)$). These terms are, therefore zero, for $\sigma' = \sigma$ and they are, for all $n \geq 2$, increasing functions in $\sigma'(A)$ and $\sigma'(B)$, respectively. To see this for the first of these two expressions,

$$\frac{1-\sigma'(A)^n}{1-\sigma'(A)} \eta - \frac{1-(1-\sigma'(A))^n}{\sigma'(A)} - \frac{c}{\alpha}$$

note that the first term in this expression is

$$r_{B\eta} = \frac{1-\sigma'(A)^n}{1-\sigma'(A)} = \sum_{k=0}^{n-1} \sigma'(A)^k,$$

and, thus, clearly increasing in $\sigma'(A)$. Analogous arguments can be made for the second term in this first expression, as well as for both terms in the second of the two expressions. This means that the first term in brackets is negative exactly when $\sigma'(A) - \sigma(A)$ is negative and is positive exactly when the latter is positive. The same argument is true for the second term in brackets. Therefore, for any $\sigma' \neq \sigma$ with $\sigma'(C) = 0$ we, indeed, have $u(\sigma, \sigma') - u(\sigma', \sigma') > 0$. Therefore, the equilibrium σ is an ESS.

F Dynamic stability

In this section we show that the class of games we here study are all stable games, in the sense of Hofbauer and Sandholm [2009]. Results in Hofbauer and Sandholm [2009] then imply that the unique symmetric Nash equilibrium that we have identified above is asymptotically stable under a large class of evolutionary (or behavioral adjustment) dynamics, that includes, for instance, also the well-known replicator dynamics of Taylor and Jonker [1978].

In order to prove that our games are stable games, we need to introduce some of the more general notation introduced by Hofbauer and Sandholm [2009]. As we have just a single populations of players, in their notation we have $p = 1$. In their notation X denotes the space of players' mixed strategy space, which in our setting is, therefore, equal to $\Delta(S)$. The payoff function in their setting is denoted by $F : X \to \mathbf{R}^k$, where k is the number of pure strategies, i.e., $k = 4$ in our setting.[2] Translated to our notation, F is, therefore, given by

$$F : \sigma \mapsto \begin{bmatrix} u(A, \sigma) \\ u(B, \sigma) \\ u(R, \sigma) \\ u(C, \sigma) \end{bmatrix}.$$

A population game $F : X \to \mathbf{R}^k$ is a stable game, as defined by Hofbauer and Sandholm [2009], if,

$$(y - x)'(F(y) - F(x)) \leq 0, for\ all\ x, y \in X$$

[2]Hofbauer and Sandholm [2009] actually use the letter n instead of k, but for our paper n is reserved to denote the number of players.

We now prove that our games are stable games in this sense. We utilize [Hofbauer and Sandholm, 2009, Example 2.9] to show that our games are *negative dominant diagonal games*, which by [Hofbauer and Sandholm, 2009, Proposition 2.7] are stable games.

To do so we need to check two conditions. First, a condition of universal *negative frequency dependence*,

$$\frac{\partial u(s,\sigma)}{\partial \sigma(s)} \leq 0 \text{ for all } s \in \{A,B,R,C\},$$

and second,

$$\left|\frac{\partial u(s,\sigma)}{\partial \sigma(s)}\right| \geq \frac{1}{2}\left(\sum_{s'\neq s}\left|\frac{\partial u(s',\sigma)}{\partial \sigma(s)}\right| + \sum_{s'\neq s}\left|\frac{\partial u(s,\sigma)}{\partial \sigma(s')}\right|\right) \text{ for all } s \in \{A,B,R,C\}.$$

We here only provide the proof that these two conditions are satisfied for $s = A$, the proof for other strategies in $S = \{A,B,R,C\}$ proceeds analogously.

Recall that,

$$\begin{bmatrix} u(A,\sigma) \\ u(B,\sigma) \\ u(R,\sigma) \\ u(C,\sigma) \end{bmatrix} = \begin{bmatrix} \alpha r_{A\eta} + (1-\alpha) r_{A\lambda} \\ \alpha r_{B\eta} + (1-\alpha) r_{B\lambda} \\ \alpha r_{B\eta} + (1-\alpha) r_{A\lambda} - c \\ \alpha r_{A\lambda} + (1-\alpha) r_{B\lambda} - c \end{bmatrix},$$

where, by Equation 2,

$$\begin{array}{rcl} r_{A\lambda} & = & \frac{1-(1-\sigma(A)-\sigma(R))^n}{\sigma(A)+\sigma(R)} \\ r_{A\eta} & = & \frac{1-(1-\sigma(A)-\sigma(C))^n}{\sigma(A)+\sigma(C)} \\ r_{B\lambda} & = & \lambda \cdot \frac{1-(1-\sigma(B)-\sigma(C))^n}{\sigma(B)+\sigma(C)} \\ r_{B\eta} & = & \eta \cdot \frac{1-(1-\sigma(B)-\sigma(R))^n}{\sigma(B)+\sigma(R)}. \end{array}$$

We, therefore, have that

$$\frac{\partial u(A,\sigma)}{\partial \sigma(A)} = \alpha \frac{\partial r_{A\eta}(\sigma)}{\partial \sigma(A)} + (1-\alpha)\frac{\partial r_{A\lambda}(\sigma)}{\partial \sigma(A)}.$$

Note that

$$\begin{array}{rcl} r_{A\lambda} & = & \frac{1-(1-\sigma(A)-\sigma(R))^n}{\sigma(A)+\sigma(R)} \\ & = & \sum_{j=0}^{n-1}(1-\sigma(A)-\sigma(R))^j, \end{array}$$

by the well-known formula for the geometric series. Thus, $\frac{\partial r_{A\lambda}(\sigma)}{\partial \sigma(A)} \leq 0$. By the same argument $\frac{\partial r_{A\eta}(\sigma)}{\partial \sigma(A)}$ is also everywhere non-positive, and, therefore,

$$\frac{\partial u(A,\sigma)}{\partial \sigma(A)} \leq 0,$$

as desired. Analogous arguments yield that the desired first condition is satisfied:

$$\frac{\partial u(s,\sigma)}{\partial \sigma(s)} \leq 0 \text{ for all } s \in \{A,B,R,C\}.$$

Let us now consider the second condition, again for $s = A$, which can be written as

$$\begin{aligned} \left|\frac{\partial u(A,\sigma)}{\partial \sigma(A)}\right| &\geq \frac{1}{2}\left(\left|\frac{\partial u(B,\sigma)}{\partial \sigma(A)}\right| + \left|\frac{\partial u(R,\sigma)}{\partial \sigma(A)}\right| + \left|\frac{\partial u(C,\sigma)}{\partial \sigma(A)}\right|\right) \\ &+ \frac{1}{2}\left(\left|\frac{\partial u(A,\sigma)}{\partial \sigma(B)}\right| + \left|\frac{\partial u(A,\sigma)}{\partial \sigma(R)}\right| + \left|\frac{\partial u(A,\sigma)}{\partial \sigma(C)}\right|\right). \end{aligned}$$

We prove this statement in two steps. Note first that

$$u(A,\sigma) + u(B,\sigma) = u(R,\sigma) + u(C,\sigma) + 2c.$$

Therefore,

$$\frac{\partial u(A,\sigma)}{\partial \sigma(A)} + \frac{\partial u(B,\sigma)}{\partial \sigma(A)} = \frac{\partial u(R,\sigma)}{\partial \sigma(A)} + \frac{\partial u(C,\sigma)}{\partial \sigma(A)}.$$

Since $\frac{\partial u(B,\sigma)}{\partial \sigma(A)} = 0$, we then have

$$\frac{\partial u(A,\sigma)}{\partial \sigma(A)} = \frac{\partial u(B,\sigma)}{\partial \sigma(A)} + \frac{\partial u(R,\sigma)}{\partial \sigma(A)} + \frac{\partial u(C,\sigma)}{\partial \sigma(A)}.$$

As all these derivatives are either negative or equal to zero, we have

$$\left|\frac{\partial u(A,\sigma)}{\partial \sigma(A)}\right| = \left|\frac{\partial u(B,\sigma)}{\partial \sigma(A)}\right| + \left|\frac{\partial u(R,\sigma)}{\partial \sigma(A)}\right| + \left|\frac{\partial u(C,\sigma)}{\partial \sigma(A)}\right|.$$

For the second step, note that $\frac{\partial r_{A\lambda}(\sigma)}{\partial \sigma(A)} = \frac{\partial r_{A\lambda}(\sigma)}{\partial \sigma(C)}$, as well as $\frac{\partial r_{A\eta}(\sigma)}{\partial \sigma(A)} = \frac{\partial r_{A\eta}(\sigma)}{\partial \sigma(R)}$. Moreover, because $\frac{\partial r_{A\lambda}(\sigma)}{\partial \sigma(R)} = \frac{\partial r_{A\eta}(\sigma)}{\partial \sigma(C)} = 0$, we have,

$$\begin{aligned} \frac{\partial u(A,\sigma)}{\partial \sigma(A)} &= \alpha \frac{\partial r_{A\eta}(\sigma)}{\partial \sigma(A)} + (1-\alpha)\frac{\partial r_{A\lambda}(\sigma)}{\partial \sigma(A)} \\ &= \alpha \frac{\partial r_{A\eta}(\sigma)}{\partial \sigma(R)} + (1-\alpha)\frac{\partial r_{A\lambda}(\sigma)}{\partial \sigma(C)} + (1-\alpha)\frac{\partial r_{A\lambda}(\sigma)}{\partial \sigma(R)} + \alpha \frac{\partial r_{A\eta}(\sigma)}{\partial \sigma(C)} \\ &= \frac{\partial u(A,\sigma)}{\partial \sigma(R)} + \frac{\partial u(A,\sigma)}{\partial \sigma(C)}. \end{aligned}$$

As $\frac{\partial u(A,\sigma)}{\partial \sigma(B)} = 0$, and again, as all derivatives are either negative or equal to zero we have

$$\left|\frac{\partial u(A,\sigma)}{\partial \sigma(A)}\right| = \left|\frac{\partial u(A,\sigma)}{\partial \sigma(B)}\right| + \left|\frac{\partial u(A,\sigma)}{\partial \sigma(R)}\right| + \left|\frac{\partial u(C,\sigma)}{\partial \sigma(B)}\right|.$$

Finally, by adding the result of the two parts, the second condition follows for $s = A$. For strategies B, R, C the same result can be proven in an analogous manner.

G Threshold Evolution

This section explores the dynamics of an evolutionary process in which individuals adopt threshold strategies. The simulation aims to capture the evolutionary changes in these strategies over generations, with selection and mutation shaping the population's behavior. We employ a replicator-dynamics-like selection process where the average payoffs (food-shares) in the game determine reproductive success. The parameter λ is fixed at 0 to avoid having strategy B as part of the equilibrium, simplifying the analysis and allowing for a more straightforward graphical representation of the evolutionary process. Individuals, thus, employ one of two possible strategies: R (responsive) or A (non-responsive). We choose $\eta = 3$. The equilibrium predictions in this case are:

$$\begin{aligned} \sigma(A) &= \tfrac{1}{4} \\ \sigma(R) &= \tfrac{3}{4} \end{aligned}$$

For the idiosyncratic preference part, as laid out in Section C, we assume that $\theta_B = 0$, $\theta_A = 0$, and θ_R is uniformly distributed on the interval $[-\epsilon, \epsilon]$ with $\epsilon = 0.1$. The first assumption ($\theta_B = 0$) is made for simplicity and the second assumption ($\theta_A = 0$) is then without loss of generality, as all that matters is the difference between θ_A and θ_R. In this example, an individual will choose the strategy R when θ is higher than their own threshold $\bar{\theta}$, otherwise the strategy A is chosen.

In our evolutionary simulations, at each point in time, we have exactly $n = 100$ individuals with a built-in threshold $\bar{\theta}$ who draw a preference type θ_R from the uniform distribution and play R if their realized $\theta_R > \bar{\theta}$. The next $n = 100$ individuals have a threshold with a probability that is proportional to how successful individuals with that threshold were in the previous period. This way the evolutionary process somewhat mimics the replicator dynamics, however, without having an infinite background population (of which these $n = 100$ are drawn each period).

The thresholds, denoted by $\bar{\theta}$, are initially drawn from a uniform distribution over the range of possible values of θ_R, $[-\epsilon, \epsilon]$. This initialization ensures diversity in the population's strategies, allowing the simulation to capture the dynamics of evolutionary adaptation.

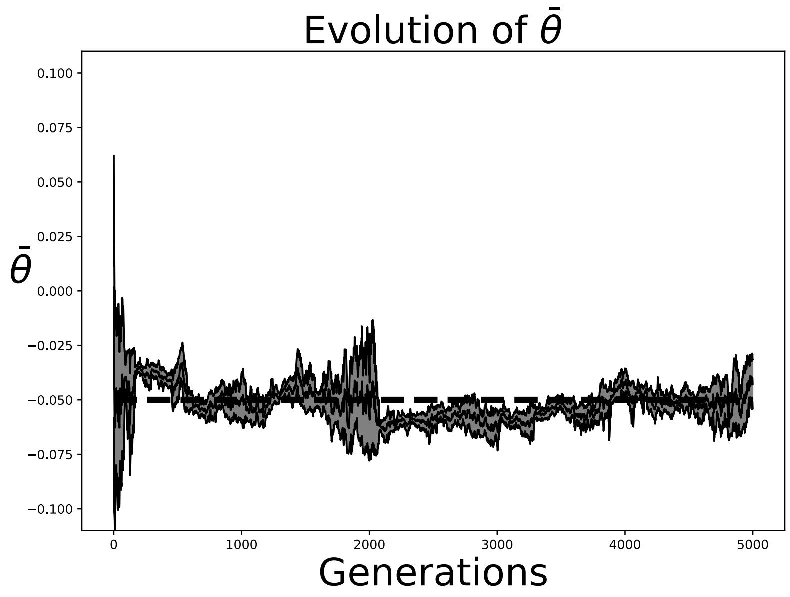

Figure 1: **Evolution of threshold strategies over generations.** Possible thresholds are on the y-axis. The x-axis tracks generational time steps. There are four lines. The dashed straight line (constantly at -0.05) is the threshold that would yield the equilibrium frequencies $\sigma(R) = \frac{3}{4}$ of responsive (if θ_R falls above -0.05) and $\sigma(A) = \frac{1}{4}$ of non-responsive behavior (if θ_R falls below -0.05). The middle one of the remaining three lines is the average threshold within the $n = 100$ individuals at each generation. The other two lines represent this average threshold plus and minus two times the standard deviation of thresholds in the population.

In each generation (or period), an individuals' average payoff is calculated as their average payoff over 10 independent realizations of θ.[3]

[3]One way to interpret this is that we assume that every individual redraws its θ 10 times in its lifetime. Under this interpretation there is little consistency of behavior. Another interpretation would be that we are here actually talking about 10 and not 1 individuals all with the same threshold and all with independently drawn preference perturbations θ_R and we consider their average reproductive success. We have also run simulations

The replication probability p_i of an individual i with threshold $\bar{\theta} \in [-\epsilon, \epsilon]$ at a given point in time is proportional to their (average) payoff, denoted by u_i, given the strategy distribution at that time, relative to the total (average) payoff in the population. Formally,

$$p_i = \frac{u_i}{\sum_{j=1}^{N} u_j}$$

Mutations occur with a probability of 0.02. When a mutation happens, it is modeled as a small appropriately truncated Gaussian additive perturbation applied to the threshold of the offspring. These perturbations are sampled from a normal distribution, $\mathcal{N}(0, \nu)$, where the mean is 0 and the standard deviation is given by ν. Such mutations introduce variability into the population, simulating random genetic drift and allowing exploration of new strategies. To ensure that the mutated threshold values remain coherent, the resulting values are constrained within predefined bounds $[-\epsilon, \epsilon]$. Any value exceeding these bounds is clipped to the nearest boundary, preventing the population from adopting threshold strategies that are outside the plausible range defined by the model's assumptions.

Figure 1 illustrates the evolution of the distribution of thresholds over time (or generations) for 5000 generations. One can see a very quick evolution of the average threshold (starting at 0) to the long-run equilibrium value around -0.05. The steady rate of mutations then implies some mutation-driven variation across individual thresholds centered around this long-run equilibrium value.

with instead of taking the average payoff over 10 realizations, taking the average payoff over 100 realizations or even just one realization per individual. The simulation results are qualitatively very similar, only the magnitude of the temporary short-run deviations around the equilibrium threshold varies somewhat.
